# Automated classification of protein expression levels in immunohistochemistry images to improve the detection of cancer biomarkers

**DOI:** 10.1186/s12859-022-05015-z

**Published:** 2022-11-08

**Authors:** Zhen-Zhen Xue, Cheng Li, Zhuo-Ming Luo, Shan-Shan Wang, Ying-Ying Xu

**Affiliations:** 1 School of Biomedical Engineering and Guangdong Provincial Key Laboratory of Medical Image Processing, Southern Medical University, Guangzhou, 510515 China; 2 Guangdong Province Engineering Laboratory for Medical Imaging and Diagnostic Technology, Southern Medical University, Guangzhou, 510515 China; 3 Paul C. Lauterbur Research Center for Biomedical Imaging, Shenzhen Institutes of Advanced Technology, Chinese Academy of Sciences, Shenzhen, 518055 China; 4 Peng Cheng Laboratory, Shenzhen, 518055 China; 5 Guangdong Provincial Key Laboratory of Artificial Intelligence in Medical Image Analysis and Application, Guangzhou, 510080 China

**Keywords:** Protein expression level, Cancer biomarker, Bioinformatics, Bioimage processing, Machine learning

## Abstract

**Background:**

The expression changes of some proteins are associated with cancer progression, and can be used as biomarkers in cancer diagnosis. Automated systems have been frequently applied in the large-scale detection of protein biomarkers and have provided a valuable complement for wet-laboratory experiments. For example, our previous work used an immunohistochemical image-based machine learning classifier of protein subcellular locations to screen biomarker proteins that change locations in colon cancer tissues. The tool could recognize the location of biomarkers but did not consider the effect of protein expression level changes on the screening process.

**Results:**

In this study, we built an automated classification model that recognizes protein expression levels in immunohistochemical images, and used the protein expression levels in combination with subcellular locations to screen cancer biomarkers. To minimize the effect of non-informative sections on the immunohistochemical images, we employed the representative image patches as input and applied a Wasserstein distance method to determine the number of patches. For the patches and the whole images, we compared the ability of color features, characteristic curve features, and deep convolutional neural network features to distinguish different levels of protein expression and employed deep learning and conventional classification models. Experimental results showed that the best classifier can achieve an accuracy of 73.72% and an F1-score of 0.6343. In the screening of protein biomarkers, the detection accuracy improved from 63.64 to 95.45% upon the incorporation of the protein expression changes.

**Conclusions:**

Machine learning can distinguish different protein expression levels and speed up their annotation in the future. Combining information on the expression patterns and subcellular locations of protein can improve the accuracy of automatic cancer biomarker screening. This work could be useful in discovering new cancer biomarkers for clinical diagnosis and research.

**Supplementary Information:**

The online version contains supplementary material available at 10.1186/s12859-022-05015-z.

## Background

Protein biomarker screening is important for the early diagnosis of cancers [1]. Protein biomarkers are usually identified by detecting the difference in protein expression between normal and cancerous tissues [2] and are generally grouped into two categories, i.e., expression biomarkers and location biomarkers [3]. The former is based on the changes in protein expression patterns, and the latter is based on the changes in protein subcellular locations. Both can reflect different disease states and have played remarkably roles in disease diagnosis. For example, protein HE4 (Human Epididymis Protein 4) is not expressed in normal surface epithelium and highly restricted in normal tissues but is overexpressed in ovarian cancer and lung adenocarcinoma cells [4–6]. GOLPH2 expression is significantly higher in prostate cancer glands than in normal glands, so this protein is regarded as a promising candidate biomarker for prostate cancer diagnosis [7]. For the location biomarkers, protein cyclin D1 is mainly localized in the cytoplasm and nucleus of normal tissues but is only detected in the nucleus of ovarian cancer cells [8]. The translocation of protein FOXO3 from nucleus to cytoplasm is associated with poor survival among patients with breast cancer [9].

The large-scale screening of cancer biomarkers has provided a critical reference for wet-laboratory, where machine learning is the core tool of automated screening [3, 10–12]. For example, Murphy’s group simultaneously considered protein expression level and subcellular location, and used the numerical features of image patches to detect difference between normal and cancerous tissues [3]. Their work simultaneously considered both factors and effectively identified biomarker proteins; however, they did not train classification models and cannot determine the exact changes of protein distribution in cancer tissues. We previously described a classification model that can recognize the subcellular location of proteins in immunohistochemical (IHC) images and detect location biomarkers [13]; however, the detection performance was largely affected by the variations of protein expression. Hence, an automated system that can comprehensively and accurately detect the changes of protein distribution is needed.

In this work, we constructed an automated classifier for that recognizes protein expression levels in IHC images, and then incorporated it with our previously built location predictor to detect cancer biomarkers. Image patches were used as input in the classifier, and a Wasserstein distance strategy was applied to determine the patch number. Color features, characteristic curve features and deep learning features were employed to describe the protein expression levels in the images and patches and then incorporated into three different classification models. Classification and application results demonstrated that the model can accurately recognize the protein expression levels and greatly improve the biomarker identification.

## Results and discussion

**Fig. 1:**
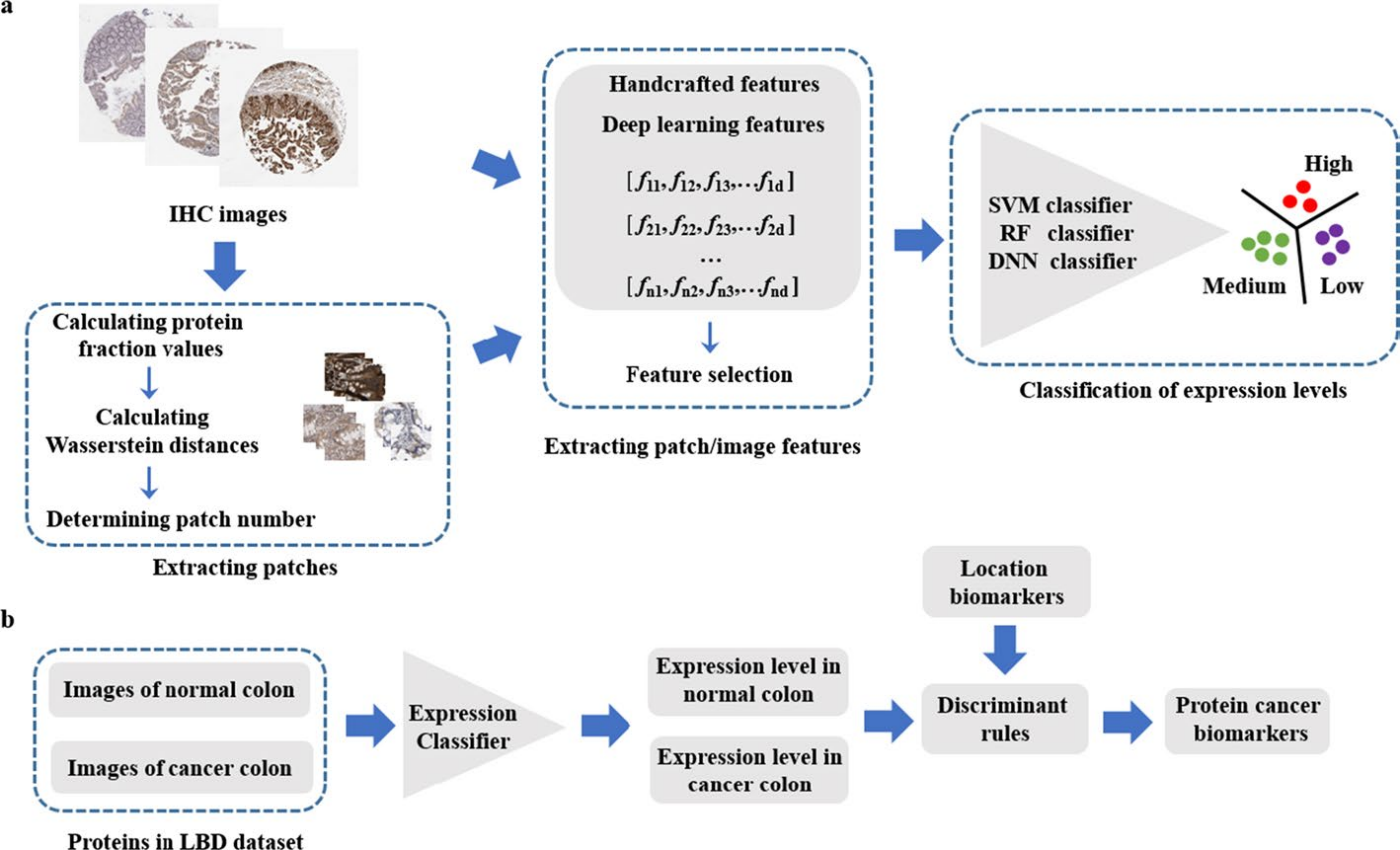
Framework of the experiments in this study

The flow chart of the experiments in this study is depicted in Fig. 1, which includes two stages, i.e., training classifiers and screening cancer biomarkers. In the first stage, we built machine learning models that can classify three protein expression levels in IHC images, i.e., high, medium, and low. The model building has three steps, i.e., extracting patches, calculating the features of patches or images, and training deep learning or conventional classification models (METHODS). In the second stage, we incorporated the best protein expression level classifier with the previous protein subcellular location predictor to test whether the screening of protein cancer biomarkers can be improved.

### Determining the patch extraction parameter

In protein expression prediction, whole IHC images are difficult to directly analyze because of their unstained stromal and unspecific backgrounds. Assuming that the highly stained cellular regions can represent the protein expression patterns of the whole images, we used a low-pass filter to slide on protein channels. We then separated these channels from the IHC images by linear spectral unmixing (LIN) to extract the square patches of interest with the highest protein expression levels. on the basis of our previous work about IHC image processing, the patch size was set to 224 × 224 pixels [13].

**Fig. 2:**
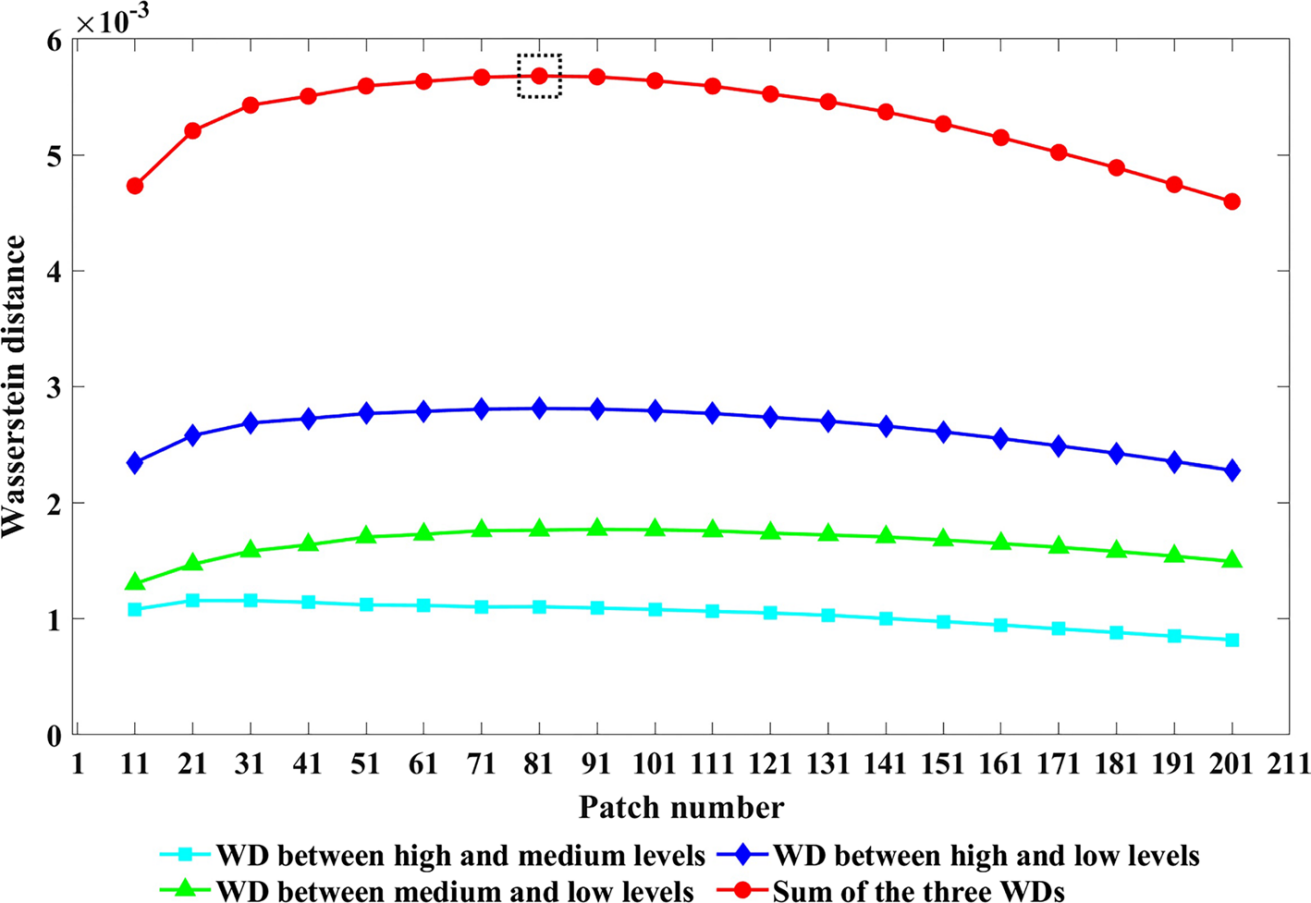
Wasserstein distances between different protein expression levels. The black dotted box represents the maximum value of the combined Wasserstein distance. WD: Wasserstein distance

To determine the number of patches extracted from each IHC image, we conducted a preliminary experiment by randomly selecting 100 proteins for each expression level from the modeling dataset to search for the patch extraction parameter. For each patch number in range [11, 21,…, 201], we calculated the protein fraction values for the extracted image patches, and used the Wasserstein distance between the distribution values of different protein expression levels to measure the classifier’s ability to distinguish the protein expression levels. We repeated the random selection of proteins for five times to reduce bias, and the average calculated distances are shown in Fig. 2.

**Fig. 3:**
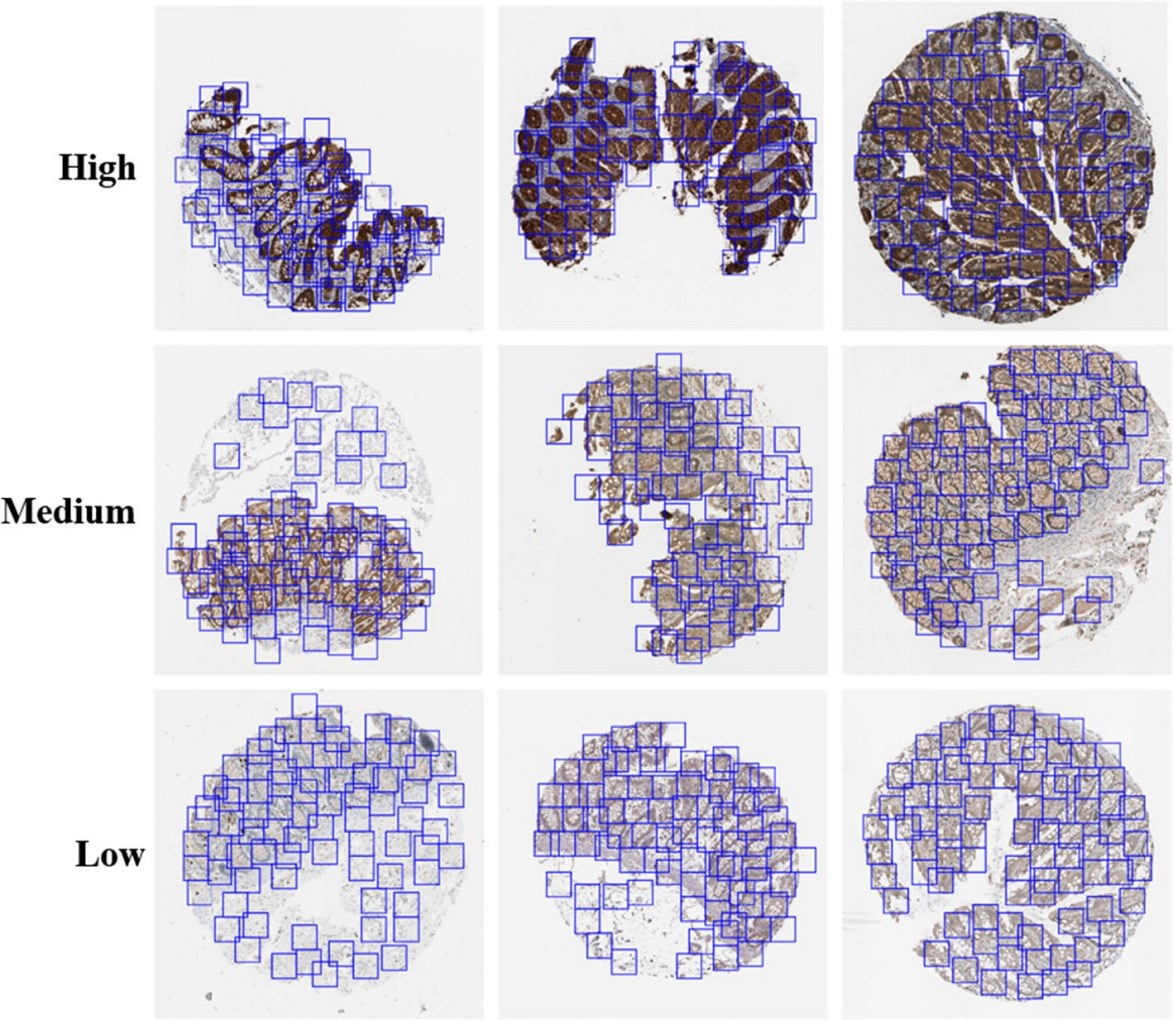
Some example IHC images and positions of extracted patches

The sum of Wasserstein distances (the red line) showed an increasing first and then decreasing trend with the increase in the number of image patches. This finding indicated that in distinguishing the expression levels in the images, sufficient patches must be extracted to obtain information. However, when the patches are excessive, overlaps would occur and lead to information redundancy and noise introduction. The results showed that the maximum Wasserstein distance can be obtained when the number of patches from one image was 81 (Fig. 3); therefore, we adopted this number in the following experiments. As illustrated Fig. 2, the distance between high and low levels was roughly equal to the sum of the distance between high and medium levels and the distance between medium and low levels. This result reflected that the Wasserstein distance method could accurately describe the difference and distance among the three expression levels.

### Investigating classification ability of statistics of protein expression

The above experiments for determining the patch parameter applied the protein fraction values of patches to represent the three expression levels. We also investigated whether these values or the intensity of the protein channels can be used to directly distinguish the expression levels.

**Fig. 4:**
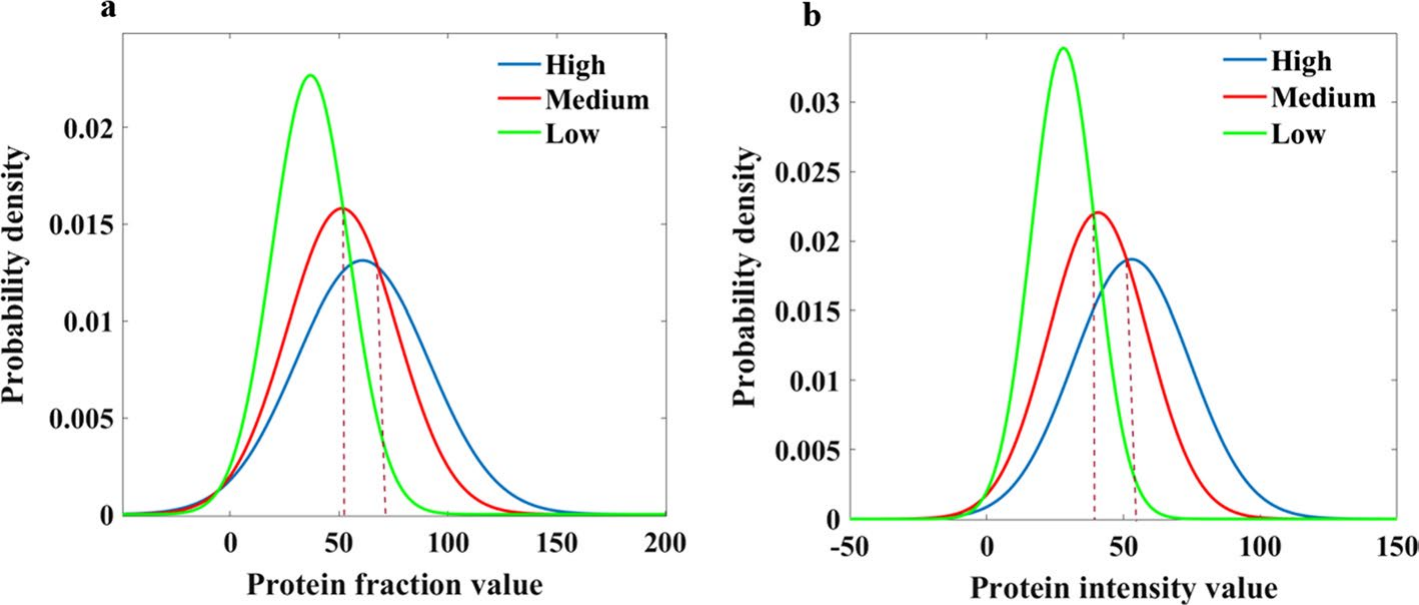
Gaussian distribution of **a** protein fraction values and **b** intensity of protein channels for high, medium and low expression levels

For the protein fraction values, we extracted 81 patches from each image in the randomly selected preliminary dataset and then calculated the protein fraction values. We fitted the protein fraction values of all image patches under each expression level with Gaussian distribution, and the Gaussian distribution results for the three levels are shown in Fig. 4a. Two thresholds can be obtained from Fig. 4a. When the protein fraction value is less than 52, we classify the patch into the low class. When the protein fraction value is greater than 52 but less than 68, we classify the patch into the medium class. When the protein fraction value is greater than 68, we classify the patch into the high class. Finally, these patches served as the basis to determine the label of the image. We applied these two thresholds to the test set of 10-fold cross validation, and the evaluation results were 37.23% for accuracy, 51.20% for recall, 45.48% for precision, and 32.89% for F1-score. For the intensity of the protein channels, we firstly removed the background of all the protein channels, and then computed the mean intensity of each channel. Gaussian distribution was also used to fit the intensities for the different protein expression levels (Fig. 4b).

The performance of classification by directly using the protein fraction values or intensity was not ideal probably because the fractions of protein regions or the color in the image patches cannot fully reflect the protein expression levels. The expression levels also considered other factors like cell types in the images. In view of the unsatisfactory classification using the two statistics, we resorted to other solutions for classification.

### Classification results obtained using the whole images and interest patches

To classify the protein expression level patterns in IHC images, we extracted 1247-dimensional handcrafted features, including color features and characteristic curve features, and deep learning features from the selected patches and the whole IHC images. The most representative features were selected by stepwise discriminant analysis (SDA), and then applied to train support vector machine (SVM), random forest (RF), and deep neural network (DNN) classifiers. The deep learning features used pre-trained networks including ResNet18, ResNet50, ResNet101, and DenseNet201. The whole IHC images and patches were fed into those networks to get feature maps. The features of patches in one image were averaged to obtain the image features, and then after the SDA step, the features were fed into the different classification models to perform 10-fold cross validations. For the DNN model, a feedforward artificial neural network was used in this experiment. The DNN network architecture was determined by searching the number of layers and the number of neurons in each layer (Additional file 1: Table S1). Finally, we employed two hidden layers with 200 neurons per layer. Given that the feature extraction step has obtained abundant information on protein expression levels from the images, the network does not need to be deep.

**Fig. 5:**
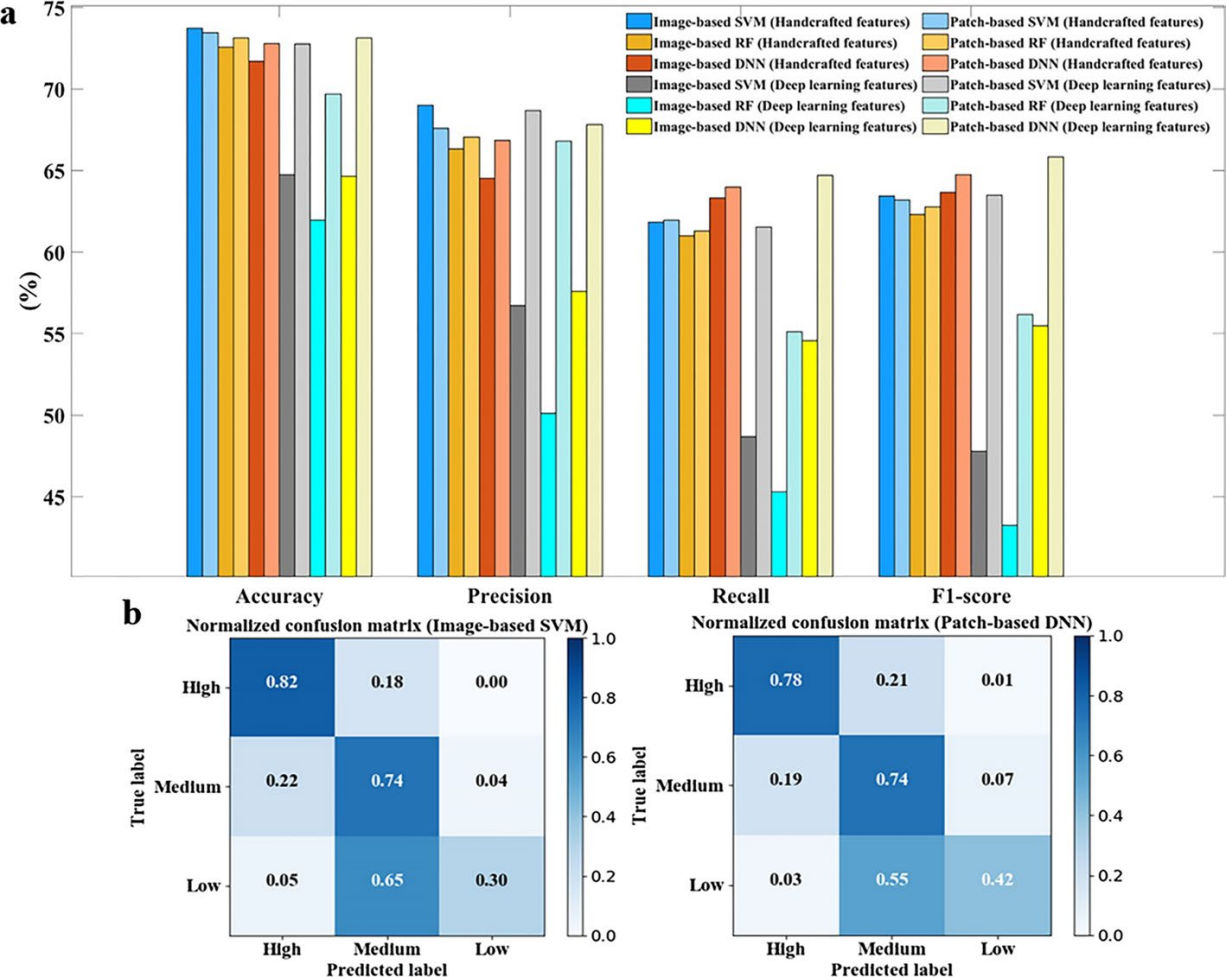
Classification results of different protein expression levels. **a** Classification results of SVM, RF, and DNN models based on images and patches. **b** Confusion matrices calculated based on results of the image-based SVM model and patch-based DNN model

Figure 5a shows the 10-fold cross validation results of the classifiers. The deep learning feature method only presented the results of the ResNet101 network, and results of other networks were shown in Additional file 1: Table S2. According to these findings, the whole IHC image-based and patch-based classifiers could distinguish different protein expression levels to some extent. Their performances were comparable, except for those based on deep learning features. The RF and DNN methods achieved a great performance when using the patches as input, and the SVM models performed well when using the image handcrafted features. But in the deep learning features from pre-trained network, The SVM, RF and DNN methods achieved higher performance when using the patches as input. It points out that the deeper layers of the network can capture the local features of the image. Considering that the protein expression levels of images are manually annotated by specialists, who tended to focus on the global image staining, rather than the local cell regions with high protein expression, we chose the image-based SVM classifier as the tool to detect protein expression changes in cancerous tissues.

The SVM models achieved the highest accuracy, and the DNN obtained the best F1-score. This inconsistency was due to the imbalance of data. In our dataset, the number of images in the high or medium class was over three times more than that in the low class; this imbalance caused many images in the low class to be misclassified into the medium class (Fig. 5b). As a consequence, the SVM models obtained high accuracy but low F1-score. Given its classification performance and simple image preprocessing, the image-based SVM classifier was still the optimal model for further application.

### Results of cancer biomarker screening

Our previous work screened cancer biomarkers using changes in the subcellular locations of proteins between normal and cancer tissues [13]. However, this method was not sensitive to the biomarkers that change their protein expression levels but do not translocate. Therefore, the protein expression patterns must be considered for a comprehensive biomarker screening. We applied the final SVM model to predict the literature biomarker dataset (LBD) and test its ability to distinguishing protein cancer biomarkers (METHODS). The LBD includes 770 images of 22 proteins in normal and cancerous colon tissues, and the accuracy of the predicted expression levels for images of normal and cancer tissues were 72.32% and 68.69%, respectively. We performed an independent sample *t*-test based on the predicted score vectors and used the *P* values to assess the significance of protein expression changes.

Table 1 shows the predicted protein expression levels and the *P* values of protein expression patterns. For comparison, we also listed the *P* values of the screened location biomarkers based on protein subcellular locations [13]. The results showed that 14 out of the 22 proteins can be detected as cancer biomarkers based on their expression levels. Comparison revealed the detection rate could be improved from 63.64% (screening using only subcellular locations) to 95.45% (considering expression level and subcellular location), that is, 21 of 22 proteins in the LBD dataset could be identified as cancer biomarkers. In particular, proteins NDRG1, BCL2 and CYSLT1 do not largely change their subcellular locations but show significant changes of expression level in cancer colons, so they can be correctly identified by our method. Hence, the combination of prediction methods for protein subcellular location and expression level could provide an effective way to screen cancer biomarkers.

**Table 1:** Results of identifying protein biomarkers

Protein	Predicted expression level change	Expression level change (*P* value)	Subcellular location change (*P* value)
BCAR1	Medium → Low	0.5437	**Nucl.: 0.0455**
ELAVL1	Medium → High	0.7435	**Cytopl.: 3.6e-5**
NDRG1	High → Medium	**3.37e-4**	PlasMem.: 0.1675 Nucl.: 0.1005
CCNEL	Medium → Low	0.0706	Nucl.: 0.8563
AHR	Medium → Low	**0.0094**	**Nucl.: 0.0158**
p68	Medium → Low	**0.0029**	**Cytopl.: 4.6e-7**
EBP50	High → Medium	0.1086	**Nucl.: 0.0076**
CDKN1B	Medium → Low	**2.14e-3**	**Cytopl.: 6.65e-8**
CACYBP	Medium → High	**2.85e-7**	**Nucl.: 1.03e-5**
ARRB1	High → Low	0.5986	**PlasMem.: 1.12e-3**
p53	High → Medium	**9.34e-3**	**Cytopl.: 0.0498** **Nucl.: 0.0365**
BRD4	High → Low	**0.0376**	**Nucl.: 1.3e-5**
AKT1	Medium → Low	**1.04e-3**	**Cytopl.: 0.0204**
PRKCA	Medium → Low	**2.13e-3**	**PlasMem.: 4.04e-11**
SMAD3	Medium → Low	**0.0225**	**Nucl.: 0.0249**
HNRNPK	High → Low	0.1058	**Cytopl.: 3.46e-4** **Nucl.: 0.0110**
PRKCB	Low → High	0.3276	**PlasMem.: 0.0240**
ß-catenin	Medium → Low	0.6826	**Cytopl.:1.65e-4** **Nucl.: 2.86e-4**
STAT3	Medium → Low	**5.88e-4**	**Nucl.: 5.2e-5**
TET2	Medium → High	**0.0491**	**Cytopl.:1.86e-3**
BCL2	Low → Medium	**0.0203**	Nucl.: 0.2644 PlasMem.: 0.1820
CYSLT1	Low → Medium	**0.0178**	Nucl.: 0.5582

The bold are the screened cancer biomarkers

## Conclusions

In this work, we proposed a method that can automatically classify the protein expression patterns in IHC images and proved that it can improve the practicability of cancer biomarker screening. The feature descriptors can be effectively used in classification to automatically score the protein expression patterns in IHC images. For cancer biomarker screening, the combined information of protein subcellular location and expression pattern can be used to precisely and efficiently identify cancer biomarkers.

At present, the research on cancer biomarkers screening requires automatic methods for improved accuracy. Our future research will focus on analyzing additional texture features representing the morphological features of membrane staining to improve the classification accuracy, and help reduce the overlap between low and medium and between high and low classes. Additionally, accurate data labeling is necessary to improve the classification performance. However, the labeling of protein expression information is a time-consuming and laborious project. The labeled data are limited and may contain labeled noise and unlabeled data. Therefore, our future work will consider using weakly supervised learning, semi-supervised learning or unsupervised learning to improve the classification of protein expression patterns.

## Methods

### Datasets

**Fig. 6:**
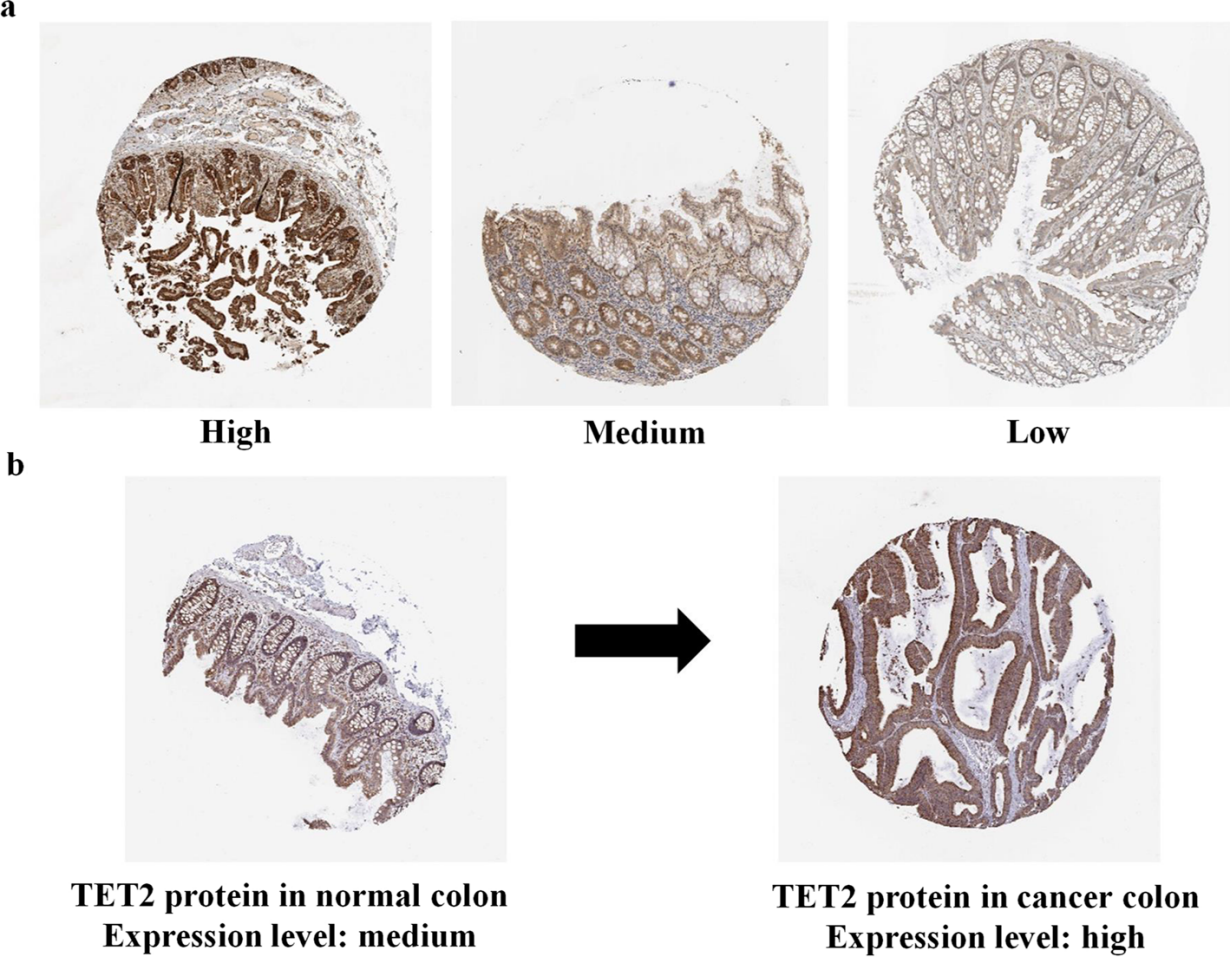
Example IHC images with different protein expression levels. **a** Example images. **b** An example of expression biomarker (TET2) for colon cancer

In this study, our image datasets were collected from the human protein atlas (HPA, https://proteinatlas.org) database, a public online database containing more than 1 million IHC microscopy images showing the distribution of proteins in healthy and cancerous human tissues [14, 15]. Each IHC image is a colored RGB image and has approximately 3000 × 3000 pixels. For quality assurance, all the images in the HPA are generated under the same imaging conditions, and the expression levels were annotated by trained experts. In the HPA, all the IHC images are manually annotated by a specialist and then verified by a second specialist to provide an overview of protein expression. Basic annotation parameters for each image include the evaluation of staining intensity (negative, weak, moderate, or strong) and fraction of stained cells (< 25%, 25–75%, or > 75%). In this work, we selected IHC images according to two criteria to ensure the quality of data: (a) staining intensity was annotated as strong or moderate, and (b) the fraction of stained cells was annotated as greater than 25%. The protein expression level of each image was annotated as high, medium, low, or not detected in the HPA. The last level means that no protein expression can be detected in the image, so we excluded the images classified this level. Therefore, the images in our datasets have three expression levels, i.e., high, medium, and low (Fig. 6).

Using the above criteria, we collected two datasets, i.e., modeling dataset and literature biomarker dataset. The former consists of 6509 IHC images of 1638 proteins in normal colon tissue, and was used to build classifiers for protein expression level patterns. The latter has 22 proteins that have been reported as cancer biomarkers, and was used to validate the performance of the models on screening biomarkers [13]. Additional details of the two datasets are shown in Table 2 (modeling dataset) and in Ref [13] (LBD).

**Table 2:** Information of the modeling dataset

Protein expression level	Number of proteins	Number of IHC images
High	573	3211
Medium	488	2675
Low	137	833

### Image preprocessing

IHC images are a major source of data in proteomics research. Each image shows a mixture of brownish diaminobenzidine staining and purple hematoxylin staining, where regions of a specific protein are stained brown, and nuclei and cell bodies are stained purple. Given that the distribution of proteins is the key factor in protein expression classification, separating the protein channels is critical in building the classifiers. Our previous work showed that LIN performed well in separating protein and DNA channels [13], so we continued to use this method to obtain the protein channels.

### Patch extraction

To determine how many patches should be extracted from one image, we searched this parameter over a range of 11–201 in steps of 10 using a small part of data containing randomly selected IHC images labeled with high, medium, and low protein expression levels. For each image, we extracted a certain number of patches according to the separated protein channel, and calculated the fraction of the areas occupied by protein in each patch. Thus, each of the three expression levels would have various fraction values, which we then fitted to a gamma distribution. The fraction value distributions of the three expression levels are expected to be different, so the criterion for searching the patch number was that the optimal number would present the largest distances among the three distributions. Here, we used Wasserstein distance to measure the distribution differences [16] because it has been widely applied and could maintain the original feature distributions. For a certain patch number, we can calculate the Wasserstein distances between protein expression patterns as described above. If the Wasserstein distance could reach the maximum, then the corresponding number of image patches would be regarded as the optimal.

### Feature extraction and selection

We extracted color features and characteristic curve features from the selected patches and the whole IHC images to build classifiers. The color features included image statistical features color histogram, color moment, color coherence vector, and color correlogram. We extracted the color histogram features to represent image color distributions. This step partitioned the underlying color spaces into a fixed number of bins, and each of color spaces corresponded to a bin in the histogram [17]. Color moment is a simple and effective color feature and uses the mean, variance, skewness, and kurtosis in RGB and HSV spaces to describe color distributions in images. In the calculation of color coherence, spatial information is first considered to classify each pixel in a given color bucket as either coherent or incoherent based on whether it is part of a large similarly colored region; the numbers of coherent and incoherent pixels with each color are then used as features [18]. Color correlogram calculates the proportion of pixels of a certain color in the whole image and reflects the spatial correlation between different color pairs [19].

We also employed a novel feature encoder called characteristic curves, as percentage of membrane staining perceived in an image is an important factor for the expression level [20]. Specifically, the images were firstly converted to the Hue-Saturation-Value (HSV) space, where [*h, s, v*] represent the stain color components in the HSV space. Then the hue and value thresholds were fixed, and only the lower bound for the saturation was specified. The *p*(*s*_*low*_) denotes the percentage of staining with color in the range given by the following inequalities:

1 $$h_{1} \le h < h_{2}$$

2 $$s > s_{low}$$

3 $$v_{1} \le v \, < \, v_{2}$$

In the process of calculating the percentage, the *p*(*s*_*low*_) was progressively increasing typically from 0.1 to 0.5. The distribution of saturation in a certain range was plotted and discretized to be the curve features. Thus, the handcrafted features were extracted for each patch and for each IHC image, including 1012 color histogram features, 33 color moment features, 54 color coherence vector features, 128 color correlogram features, and 20 characteristic curve features.

Here, we also extracted deep learning feature from pre-trained networks, i.e., ResNet18, ResNet50, ResNet101, and DenseNet201. The feature maps in the last fully connected layers of the pre-trained networks were extracted as patch features or image features. Each of the pre-trained networks produced an output of 1000 features.

Each patch or the whole image has a high-dimensional feature vector, which will inevitably lead to a disaster of dimensionality if the features are directly fed into classifiers. Therefore, we used a feature selection method, i.e., SDA, which has been proved to be effective in this field [21], to reduce the feature dimensionality.

### Building classifiers

To construct the protein expression level classifiers, we employed SVM, RF, and DNN. We the performed 10-fold cross validation and divided the training and test sets at the protein level. One protein usually has multiple images, and the protein-level division would ensure that all the images from the same protein are either in the training set or in the test set. In each fold experiment, we fed the features selected by SDA into SVM (LIBSVM-3.23 toolbox, https://www.csie.ntu.edu.tw/~cjlin/libsvm/) and RF models to train the classifiers, where the model parameters *g* and *c* of SVM and the number of trees in RFs were determined by grid search. We also constructed a simple DNN model to classify the protein expression levels. The network contains two hidden layers, and each fully connected layer contains 200 neurons. In addition, the network uses Adam optimizer and a mean square error loss function to optimize the weights. We performed 10-fold cross on the network to obtain achievable performance estimates, with each fold being trained for 200 epochs.

### Screening cancer biomarkers

We explored the performance of the automated model in screening cancer biomarkers in LBD. In this study, we utilized the constructed protein expression level classifiers to identify protein cancer biomarkers, and combined the results with the translocation biomarkers. Assuming that a protein has *n* images of normal colon and *m* images of colon cancer tissue, the expression patterns of this protein in normal and cancer colons were separately determined by voting based on the prediction outputs of images. We measured the expression changes by conducting an independent sample *t*-test for each protein between the *n* outputted probability vectors of the normal tissue images and the *m* vectors of the cancer tissue images. The *t*-test would output a *P *value representing significant expression change. A protein was considered to be an expression biomarker only when its *P *value was less than 0.05. These expression biomarkers served as supplementary to the location biomarkers detected in our previous work.

## Supplementary Information

**Additional file 1**.** Table S-1**. Comparison of different architectures of deep neural networks.** Table S-2**. Results of deep learning features.

## Data Availability

The datasets are collected from https://proteinatlas.org, and the codes used in this study are available at https://github.com/zzxue-08/IHC-protein-expression-level.

## Abbreviations

IHC: Immunohistochemistry
HPA: Human Protein Atlas
LBD: Literature biomarker dataset
LIN: Linear spectral unmixing
SDA: Stepwise discriminant analysis
SVM: Support vector machine
RF: Random forest
DNN: Deep neural network
