# Supplementary material for: Automated classification of protein expression levels in immunohistochemistry images to improve the detection of cancer biomarkers
Source: BMC Bioinformatics. 2022 Nov 8;23:470. doi: 10.1186/s12859-022-05015-z (PMC9644510; doi:10.1186/s12859-022-05015-z)
Supplement: Supplementary file 1 — Additional file 1. Table S-1. Comparison of different architectures of deep neural networks. Table S-2. Results of deep learning features. [file 12859_2022_5015_MOESM1_ESM.docx]

**Supplementary Material for**

Automated classification of protein expression levels in immunohistochemistry images to improve the detection of cancer biomarkers

Zhen-Zhen Xue ^1, 2, 3^, Cheng Li ^3^, Zhuo-Ming Luo ^1, 2^, Shan-Shan Wang ^3, 4, 5^ and Ying-Ying Xu ^1, 2,^*

^1^ School of Biomedical Engineering and Guangdong Provincial Key Laboratory of Medical Image Processing, Southern Medical University, Guangzhou 510515, China

^2^ Guangdong Province Engineering Laboratory for Medical Imaging and Diagnostic Technology, Southern Medical University, Guangzhou 510515, China

^3^ Paul C. Lauterbur Research Center for Biomedical Imaging, Shenzhen Institutes of Advanced Technology, Chinese Academy of Sciences, Shenzhen 518055, China

^4^ Peng Cheng Laboratory, Shenzhen 518055, China

^5^ Guangdong Provincial Key Laboratory of Artificial Intelligence in Medical Image Analysis and Application, Guangzhou 510080, China

**Corresponding author**

*E-mail: [yyxu@smu.edu.cn](mailto:yyxu@smu.edu.cn)

Tel.: +86-020-62789343

**Table S-1.** Comparison of different architectures of deep neural networks. Ten-fold cross validation results are shown.

| Network architecture | | Patch-based model | | | | | Image-based model | | | |
| --- | --- | --- | --- | --- | --- | --- | --- | --- | --- | --- |
| #. layers | #. neurons | F1-score | Accuracy | Precision | Recall | F1-score | | Accuracy | Precision | Recall |
| 2 | 50 | 62.77% | 73.45% | 67.37% | 61.43% | 62.54% | | 72.40% | 65.66% | 61.32% |
| 2 | 100 | 64.77% | 73.40% | 67.58% | 63.71% | 62.63% | | 71.41% | 64.00% | 62.01% |
| **2** | **200** | **64.74%** | **72.79%** | **66.86%** | **63.97%** | **63.65%** | | **71.70%** | **64.53%** | **63.32%** |
| 2 | 400 | 64.03% | 72.28% | 66.15% | 63.28% | 63.09% | | 71.20% | 63.83% | 62.84% |
| 2 | 600 | 63.73% | 72.56% | 66.14% | 62.83% | 62.46% | | 70.79% | 63.42% | 62.11% |
| 2 | 800 | 63.99% | 72.60% | 66.81% | 63.23% | 62.87% | | 70.98% | 63.90% | 62.58% |
| 2 | 1000 | 62.02% | 71.42% | 64.80% | 61.13% | 62.56% | | 71.05% | 63.87% | 62.26% |
| 1 | 200 | 62.61% | 73.11% | 66.83% | 61.58% | 62.16% | | 71.44% | 64.42% | 61.36% |
| 3 | 200 | 63.68% | 72.72% | 66.96% | 62.47% | 62.41% | | 71.64% | 66.14% | 61.38% |
| 4 | 200 | 62.66% | 72.82% | 66.77% | 61.30% | 60.98% | | 71.24% | 64.61% | 59.78% |
| 5 | 200 | 62.84% | 72.42% | 67.26% | 61.81% | 62.20% | | 71.39% | 65.42% | 61.03% |

**Table S-2.** Results of deep learning features.

| Model | Pre-trained network | Accuracy | | F1-Score | Precision | Recall |
| --- | --- | --- | --- | --- | --- | --- |
| Patch-based SVM | ResNet18 | 72.45% | 61.86% | | 68.13% | 60.15% |
|  | ResNet50 | 71.90% | 61.29% | | 67.40% | 59.61% |
|  | **ResNet101** | **72.76%** | **63.48%** | | **68.68%** | **61.54%** |
|  | DenseNet201 | 71.73% | 60.84% | | 66.47% | 59.25% |
| Image-based SVM | ResNet18 | 62.70% | 44.14% | | 54.43% | 46.09% |
|  | ResNet50 | 63.74% | 46.12% | | 55.78% | 47.48% |
|  | **ResNet101** | **64.74%** | **47.78%** | | **56.72%** | **48.69%** |
|  | DenseNet201 | 61.68% | 44.02% | | 50.09% | 45.61% |
| Patch-based RF | ResNet18 | 68.12% | 52.50% | | 63.38% | 52.41% |
|  | ResNet50 | 69.85% | 54.56% | | 65.73% | 54.17% |
|  | **ResNet101** | **69.69%** | **56.17%** | | **66.81%** | **55.10%** |
|  | DenseNet201 | 68.95% | 53.01% | | 64.81% | 52.94% |
| Image-based RF | ResNet18 | 60.41% | 42.41% | | 53.27% | 44.24% |
|  | ResNet50 | 62.21% | 43.87% | | 50.97% | 45.73% |
|  | **ResNet101** | **61.95%** | **43.27%** | | **50.12%** | **45.30%** |
|  | DenseNet201 | 59.97% | 41.46% | | 42.59% | 43.63% |
| Patch-based DNN | ResNet18 | 72.55% | 65.81% | | 66.39% | 65.55% |
|  | ResNet50 | 73.67% | 67.09% | | 69.54% | 66.12% |
|  | **ResNet101** | **73.12%** | **65.83%** | | **67.81%** | **64.69%** |
|  | DenseNet201 | 72.38% | 65.74% | | 67.44% | 64.95% |
| Image-based DNN | ResNet18 | 60.94% | 51.51% | | 52.64% | 50.99% |
|  | ResNet50 | 63.44% | 54.64% | | 56.09% | 53.97% |
|  | **ResNet101** | **64.66%** | **55.47%** | | **57.58%** | **54.56%** |
|  | DenseNet201 | 61.15% | 52.18% | | 53.93% | 51.33% |
